# Natural killer T cells contribute to the control of acute retroviral infection

**DOI:** 10.1186/s12977-017-0327-8

**Published:** 2017-01-26

**Authors:** Elisabeth Littwitz-Salomon, Simone Schimmer, Ulf Dittmer

**Affiliations:** Institute for Virology of the University Hospital Essen, University of Duisburg-Essen, Hufelandstr. 55, 45147 Essen, Germany

**Keywords:** Retroviral infection, Natural killer T cells, Friend retrovirus, α-Galactosylceramide, Antiviral function

## Abstract

**Background:**

Natural killer T cells (NKT cells) play an important role in the immunity against viral infections. They produce cytokines or have direct cytolytic effects that can restrict virus replication. However, the exact function of NKT cells in retroviral immunity is not fully elucidated. Therefore, we analyzed the antiretroviral functions of NKT cells in mice infected with the Friend retrovirus (FV).

**Results:**

After FV infection numbers of NKT cells remained unchanged but activation as well as improved effector functions of NKT cells were found. While the release of pro-inflammatory cytokines was not changed after infection, activated NKT cells revealed an elevated cytotoxic potential. Stimulation with α-Galactosylceramide significantly increased not only total NKT cell numbers and activation but also the anti-retroviral capacity of NKT cells.

**Conclusion:**

We demonstrate a strong activation and a potent cytolytic function of NKT cells during acute retroviral infection. Therapeutic treatment with α-Galactosylceramide could further improve the reduction of early retroviral replication by NKT cells, which could be utilized for future treatment against viral infections.

**Electronic supplementary material:**

The online version of this article (doi:10.1186/s12977-017-0327-8) contains supplementary material, which is available to authorized users.

## Findings

Natural killer T cells (NKT cells) are innate-like T lymphocytes, which recognize glycolipid antigens presented by the non-classical major histocompatibility complex (MHC) class I-like molecule CD1d. NKT cells express markers, which are associated with the T cell (αβ T cell receptor) as well as the NK cell (e.g. NK cell activating C-type lectin NK1.1) lineage. They can be divided into type I (invariant or classical) and type II (non-classical) NKT cell subsets dependent on the expression of the invariant Vα14-Jα18 gene segment in mice or Vα24-Jα18 receptor in humans [1]. Activation of NKT cells occur in the absence of prior foreign antigen priming [2, 3]. For their activation several pathways are feasible such as direct stimulation via CD1d-presented lipids and/or in combination with the cytokines Interleukin (IL)-12, IL-18 as well as type I interferons (IFNs) or only cytokine-mediated activation without T cell receptor signaling [4]. NKT cells reveal important immunoregulatory functions by massive release of T helper (Th) 1 or Th2 cytokines. Thus, NKT cells activate and recruit several other cell types including NK cells, T cells, B cells, dendritic cells and neutrophils [5, 6]. In addition, they can kill infected or transformed cells through Fas-FasL mediated apoptosis and/or the perforin/granzyme exocytosis pathway [7, 8]. Engagement of the death receptor Fas by FasL results in apoptosis mediated by caspase activation [9].

NKT cells are essential for the containment of bacterial, parasites, fungal pathogens, cancer, and also viral infections. The importance of NKT cells during viral infections becomes clear given that several viruses like Lymphocytic Choriomeningitis Virus (LCMV), Cytomegalovirus (CMV), vesicular stomatitis virus, vaccinia virus, Herpes Simplex Virus (HSV)-1 and Human Immunodeficiency Virus (HIV)-1 disrupt CD1d expression on infected target cells to evade antiviral effects of NKT cells [10–13]. In those studies, mainly IFNγ production by NKT cells was analyzed. However, the exact role of NKT cells during retroviral infection is not known so far.

The Friend virus (FV) mouse model can be utilized to analyze and therapeutically modulate the function of NKT cells during acute retroviral infection in vivo. We and others have previously shown that NK cells play an important role in innate FV immunity [14–16], but NKT cells were not studied so far. FV inoculation into mice leads to infection of erythroid precursor cells as well as granulocytes and B cells [17]. FV consists of two components: the spleen focus forming virus (SFFV) and the Friend murine leukemia virus (F-MuLV). SFFV represents the pathogenic but replication-defective part of the viral complex whereas F-MuLV is replication-competent but apathogenic [18]. Infection of C57BL/6 mice results only in mild splenomegaly, but high dose infection facilitates establishment of a chronic infection. In FV-infected mice, the highest viral loads are found in the bone marrow and spleen, so we analyzed these two organs after acute FV infection [19]. Here, we demonstrate the activation and anti-retroviral efficacy of NKT cells during acute FV infection. Furthermore, we elucidated the potential role of NKT cells for immunotherapy of retrovirus infections.

### NKT cells became activated during initial FV infection

**Fig. 1:**
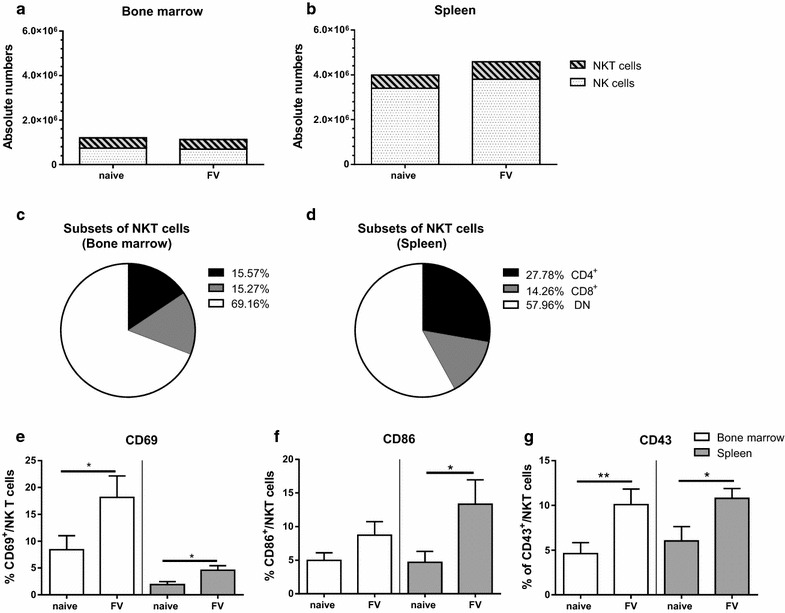
Absolute cell numbers and activation of NKT cells. Mice were infected with FV and bone marrow and spleen cells were harvested at 3 dpi. Cells were isolated and stained for NK cells (CD3^−^NK1.1^+^CD49b^+^, *dotted bars*) and NKT cells (CD3^+^NK1.1^+^, *shaded*). Absolute numbers of NK1.1^+^ cells are displayed in **a** (bone marrow) and **b** (spleen). The percentages of CD4^+^ (*black bars*), CD8^+^ (*gray bars*) and double-negative (DN, *white bars*) subsets of NKT cells in FV-infected mice were displayed in **c** (bone marrow) and **d** (spleen). NKT cells were analyzed for the activation markers CD69 (**e**), CD86 (**f**) and CD43 (**g**) using flow cytometry. In **e**, **f** and **g**, mean percentage (±SEM) of bone marrow cells are depicted with *white bars* whereas splenocytes are displayed in *gray bars*. A minimum of five mice per group were used. Experiments were repeated at least five times. Statistically significant differences between naïve and FV-infected mice were determined by the Mann–Whitney test and are indicated by *single asterisk* for *p* < 0.05 and *double asterisk* for *p* < 0.01

In some viral infections, the NKT cell population is depleted early after infection [20–22]. To analyze changes of the NK1.1^+^ cell population during initial FV infection (3 days post infection (dpi)), we first analyzed the absolute numbers of NK cells (CD3^−^NK1.1^+^CD49b^+^) and NKT cells (CD3^+^NK1.1^+^) in the bone marrow (Fig. 1a) and the spleen (Fig. 1b). Absolute numbers of NK1.1^+^ cells were around three times higher in the spleen in comparison to the bone marrow. Mainly NK cells but not NKT cells accounted for this difference in numbers. After FV infection no significant difference between the groups of naïve and the FV-infected mice was detectable, indicating that infection did not expand or diminish the NKT cell population. NKT cells are dependent on the MHC class I-like CD1d glycoprotein but only type I NKT cells respond to α-Galactosylceramide (αGalCer) stimulation [23]. Therefore, we stained cells with an αGalCer pre-loaded CD1d tetramer to identify type I NKT cells and detected very similar percentages for NKT cells (CD3^+^NK1.1^+^) and invariant NKT cells (CD3^+^ αGalCer pre-loaded CD1d tetramer^+^; Additional file 2: Figure S2 A) after FV infection. We also characterized NKT cell subsets based on their expression of CD4 and CD8. During FV infection NKT cells showed a predominant double-negative (DN) phenotype (Fig. 1c, d). Compared to naïve NKT cells, CD4^+^ and CD8^+^ NKT cell subsets slightly expanded during FV infection, while the DN population was diminished (data not shown). After FV infection the activation of NKT cells, measured by the expression of the activation markers CD69 (Fig. 1e, Additional file 1: Figure S1), CD86 (Fig. 1f, Additional file 1: Figure S1) and CD43 (Fig. 1g, Additional file 1: Figure S1), was significantly enhanced. For most activation markers the percentage of positive NKT cells was 2–3 times higher after FV infection in both analyzed organs. Similarly, we detected an enhanced activation of invariant NKT cells (CD3^+^ αGalCer pre-loaded CD1d tetramer^+^) after FV infection in both organs compared to invariant NKT cells from naïve mice (Additional file 2: Figure S2 B).

Although we could not detect differences in total NKT cell numbers, we detected a more activated phenotype of NKT cells in FV-infected mice.

### Cytokine production by NKT cells during initial FV infection

**Fig. 2:**
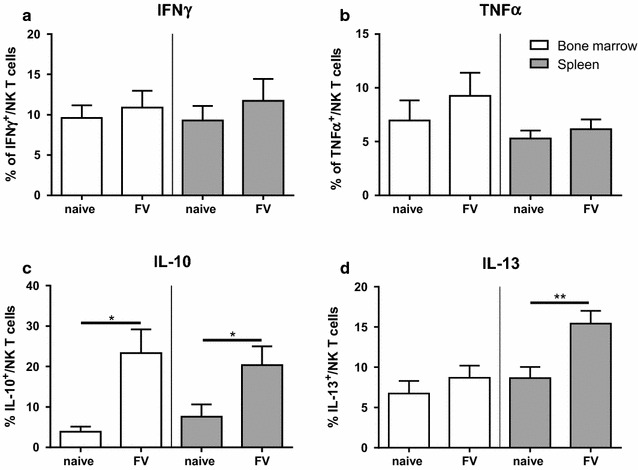
Cytokine production by NKT cells after FV infection. Bone marrow cells (*white bars*) and splenocytes (*grey bars*) were isolated from naïve or FV-infected mice (3 dpi). Cells were stimulated and stained for the pro-inflammatory cytokines IFNγ (**a**) and TNFα (**b**) and the anti-inflammatory cytokines IL-10 (**c**) and IL-13 (**d**). Mean (±SEM) values are indicated by *bars*. At least nine animals per group out of at least six experiments were used for analysis. Differences between naïve and FV-infected mice were analyzed using the Mann–Whitney test and are indicated by *single asterisk* for *p* < 0.05 and *double asterisk* for *p* < 0.01

NKT cells can produce a variety of Th1 or Th2 cytokines resulting in immunity or immune suppression. We analyzed pro-inflammatory cytokines like IFNγ (Fig. 2a) and tumor necrosis factor (TNF) α (Fig. 2b) as well as anti-inflammatory cytokines such as IL-10 (Fig. 2c) and IL-13 (Fig. 2d). We did not observe increased IFNγ or TNFα production by NKT cells post FV infection, whereas we detected significant higher percentages of NKT cells producing anti-inflammatory cytokines. In comparison to the naïve group, six-times more IL-10 producing cells were found in the bone marrow and three-times more in the spleen at 3 dpi. Also the percentage of IL-13^+^ NKT cells was significantly increased in the spleen post FV infection.

Thus, acute FV infection seems to induce the production of anti-inflammatory but not pro-inflammatory cytokines in NKT cells.

### Acute FV infection enhanced the cytotoxic potential of NKT cells

**Fig. 3:**
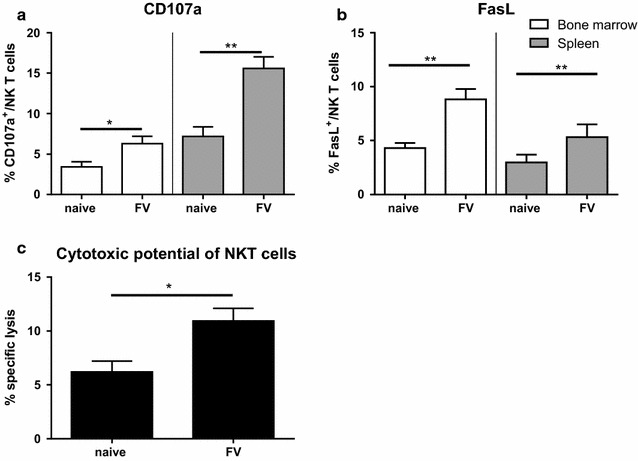
Cytotoxicity of NKT cells after FV infection. Mice were infected with FV and were sacrificed at 3 dpi. As control group non-infected mice were used. Single cell suspensions were prepared from bone marrow (*white bars*) and spleens (*gray bars*) of mice. Effector functions were measured by the degranulation marker CD107a (**a**) and apoptosis-inducing FasL (**b**) and analyzed by flow cytometry. Data were collected from at least five independent experiments and at least eight animals per group. In **c**, NKT cells from naïve and FV-infected mice were isolated and splenic and bone marrow NKT cells were mixed. NKT cells were co-incubated with CFSE-labeled, FV-transformed tumor cells (FBL-3 cells). Cells were stained for viability and immediately analyzed using flow cytometry. At least four animals per group out of at least two experiments were used for analysis. Mean (±SEM) values of percentages are indicated by bars. Statistically significant differences between groups were analyzed with the Mann–Whitney test and are indicated by *single asterisk* for *p* < 0.05 and *double asterisk* for *p* < 0.01

For the efficient containment of many virus infections effector functions from cytotoxic cells are necessary. NKT cells are competent cytokine producers but also known for their direct cytotoxic activity against virus-infected cells [7, 8]. To investigate the cytotoxic potential of NKT cells after FV infection, we analyzed the expression of degranulation marker CD107a (lysosomal-associated membrane protein-1 (LAMP-1), Fig. 3a, Additional file 1: Figure S1) associated with the release of granzyme and perforin from cytotoxic granula and FasL (Fig. 3b, Additional file 1: Figure S1) [24]. In both analyzed organs, we detected an increase in the percentage of CD107a and FasL expressing NKT cells after FV infection, which was statistically significant for both organs. In the spleen, we detected around 7% CD107a^+^ NKT cells in naïve mice and up to 16% in mice acutely infected with FV. A more than two fold higher percentage of FasL expressing NKT cells in the bone marrow was measured in FV-infected mice in comparison to naïve mice. We further analyzed whether this increase in the expression of surrogate markers for cytotoxicity was associated with enhanced cytolytic activity of NKT cells after FV infection. Indeed, co-incubation of NKT cells isolated from FV-infected mice with the FV-transformed tumor cell line FBL-3 showed a significant increase of FBL-3 cell elimination when compared to NKT cells isolated from naïve mice (Fig. 3c).

The data demonstrates that acute FV infection enhances the ability of NKT cells to kill FV-transformed target cells.

### Cytokine production and cytotoxicity of NKT cell sub-populations during early FV infection

During acute FV infection we revealed a Th2-like cytokine profile but at the same time markers of cytotoxicity in NKT cells. We were wondering if these contradictory functions were mediated by different NKT sub-populations. Therefore, we determined the proportions of CD4^+^, CD8^+^ and DN subsets from NKT cells producing pro-inflammatory and anti-inflammatory cytokines, as well as cytotoxic molecules (Table 1). The production of IL-10 and IL-13 was mainly associated with CD4^+^ NKT cells, whereas IFNγ and TNFα were mainly produced by DN NKT cells. The analysis of CD107a^+^ and FasL^+^ NKT cells and their subset distribution identified the DN NKT cells in the spleen and bone marrow as the predominantly cytotoxic NKT cell population (Table 1).

**Table 1:** Cytokine production and cytotoxicity of NKT cell sub-populations during early FV infection

	Bone marrow			Spleen		
	CD4^+^	CD8^+^	DN	CD4^+^	CD8^+^	DN
IFNγ	17 ± 6	12 ± 9	70 ± 9	24 ± 5	7 ± 6	67 ± 10
TNFα	29 ± 8	13 ± 7	51 ± 19	35 ± 7	11 ± 8	46 ± 15
IL-10	46 ± 6	11 ± 10	37 ± 6	64 ± 9	3 ± 3	26 ± 8
IL-13	50 ± 9	2 ± 1	37 ± 7	65 ± 10	3 ± 3	27 ± 11
CD107a	24 ± 4	6 ± 5	64 ± 11	23 ± 11	11 ± 6	58 ± 17
FasL	8 ± 6	12 ± 7	76 ± 8	13 ± 12	20 ± 12	55 ± 15

Cytokine production and effector functions of NKT cell subsets were analyzed by flow cytometry. Mean values and standard deviation were calculated from at least nine values out of three independent experiments. Outliers were identified with the Rout method and removed

*DN* double-negative

These results suggest different functions of NKT cell sub-populations, with CD4^+^ NKT cells mainly producing anti-inflammatory cytokines, whereas DN NKT cells express molecules associated with cytotoxicity.

### Antiviral effect of NKT cells in vivo and therapeutic stimulation of NKT cells during FV infection

**Fig. 4:**
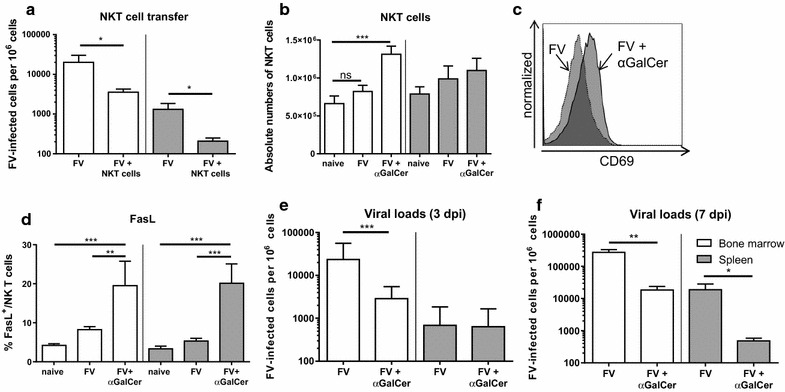
Antiviral activity of NKT cells and NKT cell activating therapy. Mice were infected with FV and splenocytes as well as bone marrow cells were used for adoptive transfer experiments. NKT cells were isolated and 1 × 10^5^ NKT cells were transferred i.v. into acutely FV-infected mice (**a**). At 3 dpi, viral loads were determined in the recipient mice. At least four mice from two different experiments were used. In **b**–**f**, one group of mice was injected with αGalCer at 0 dpi (FV + αGalCer) for stimulation of NKT cells. Absolute numbers of NKT cells per organ are shown in **b**. A representative histogram of the NKT cell activation of FV-infected mice after αGalCer stimulation is displayed in **c**. Effector function were measured by the apoptosis-inducing FasL and analyzed by flow cytometry. Data were collected from at least three independent experiments. At least eight animals per group were used for analysis. Viral loads after αGalCer treatment were examined by infectious centers assay at 3 dpi (**e**) and 7 dpi (**f**). Mean (±SEM) values of percentages are indicated by *bars*. A minimum of nine mice out of three independent experiments (**b**, **d**, **e**) or at least four mice from two different experiments were used for **f**. Statistically significant differences between groups were analyzed with the Mann–Whitney test (**a**, **e**, **f**) or the Kruskal–Wallis test (**b**, **d**) and are indicated by *single asterisk* for *p* < 0.05; *double asterisk* for *p* < 0.01 or *triple asterisk* for *p* < 0.001. *ns* not significant

Our current results show that acute FV infection activates NKT cells to produce anti-inflammatory cytokines, but at the same time enhances their cytotoxic potential. It was therefore of interest if these cells would increase or reduce FV loads in vivo. To analyze this we performed an adoptive transfer experiment with NKT cells from FV-infected mice into acutely FV-infected mice and subsequently determined their viral loads. In bone marrow and spleen, a significant decrease of more than 80% in the viral burden was detected post transfer of NKT cells (Fig. 4a), indicating that the virus-activated NKT cells mediated anti-retroviral effects in vivo. In the 1990s, αGalCer was identified as an exogenous activator for CD1d-restricted NKT cells [25]. First, it was isolated from extracts of a marine sponge but in 1995 a synthetic analogue called KRN 7000 was identified [26]. We used this compound to therapeutically stimulate NKT cells during an acute FV infection. In the bone marrow of FV-infected mice, treatment with the immunomodulatory αGalCer (KRN 7000) led to increased NKT cell numbers (Fig. 4b, Additional file 2: Figure S2 C) and augmented their activation (Fig. 4c). FasL expression by NKT cells was significantly increased in FV-infected and αGalCer-treated mice (Fig. 4d), but treatment of naïve mice with αGalCer did not result in any increase in FasL expression (data not shown). NKT cell stimulation in naïve mice slightly increased the production of anti-inflammatory cytokines but no increase in IFNγ was detected (data not shown). However, we found an augmented IFNγ production by NKT cells in the FV-infected and αGalCer-treated group of mice similar to the increased FasL expression (data not shown, Fig. 4d). At 3 dpi, we detected a mean viral titer of 23542 FV-infected cells per million cells in the bone marrow, whereas the viral loads in FV-infected αGalCer treated mice were only around 2875 FV-infected cells per million cells (Fig. 4e). Thus, the stimulation of NKT cells resulted in an 87.8% reduction of viral loads, which correlated with the expansion, activation and FasL expression of NKT cells in this organ (Fig. 4b–d). We also analyzed the effect of αGalCer therapy at a later time point and detected a more than one log reduction in viral loads at 7 dpi in the spleen and bone marrow due to the treatment (Fig. 4f). Taken together, FV-activated NKT cells mediated anti-retroviral effects in vivo and therapeutic activation of NKT cells can improve the control of acute FV infection.

Stimulation with αGalCer also led to NK cell (CD3^−^CD49b^+^NK1.1^+^) activation and cytokine production. We therefore analyzed the expression of CD69 on NK cells and their production of pro-inflammatory cytokines in FV-infected mice after αGalCer administration (Additional file 2: Figure S2 D). We detected an activation of NK cells post FV infection, which was significantly enhanced post αGalCer therapy (Additional file 2: Figure S2 D, CD69, black bars). The αGalCer treatment also increased the percentages of TNFα produced by NK cells (Additional file 2: Figure S2 D, gray bars). IFNγ production by NK cells was induced by FV infection, but was not further enhanced post αGalCer administration (Additional file 2: Figure S2 D, white bars). Thus, secondary effects of NKT cell stimulation on NK cells may partly contribute to the anti-retroviral effects after αGalCer therapy.

In this report, we analyzed the impact of NKT cells on the control of viral replication during initial phase of acute FV infection (3 dpi). We could demonstrate cytotoxicity of activated NKT cells and anti-retroviral activity in vivo. Most importantly, antiviral functions of NKT cells could be further increased by glycolipid αGalCer therapy that resulted in approximately 90% reduction in viral loads.

Various functions of NKT cells were also described in other viral infections. For example, increased numbers of NKT cells were detected in the lungs of influenza A virus (IAV) infected mice and the survival rate of NKT knockout mice after IAV infection was reduced [27, 28]. In these studies, the activation of NKT cells correlated with the reduction of IAV replication and reduced weight loss of mice [27]. Furthermore, NKT cells decreased immunopathology during IAV infection by reducing the accumulation of inflammatory monocytes in the lung [29]. In HIV infection NKT cell responses are difficult to analyze because functions of NKT cells are impaired and HIV infection results in loss of NKT cells within the first year of infection [30–32]. The initiation of antiretroviral therapy (ART) in HIV-infected individuals results in a slow recovery of circulating NKT cell subsets and improves their functionality [31, 32]. Recently it was shown that NKT cells can directly recognize and respond specifically to HIV-1-infected DCs [33]. In this study, NKT cell sensing of HIV-infected cells depends on the expression of the CD1d molecule and the presentation of endogenous lipid antigen, which is at least partially downregulated by the accessory proteins Nef and Vpu [33]. HIV is closely related to SIV that causes AIDS in macaques and serves as a well-accepted primate model for HIV infection [34]. A study in SIV-infected macaques that develop AIDS versus SIV-infected sooty mangabeys that are disease resistant, revealed a hypofunction of NKT cells in SIV-infected macaques [35]. The authors concluded that NKT dysfunction may play a role in AIDS pathogenesis and that immunoregulatory NKT cells might prevent generalized immune activation and immunodeficiency [35]. During acute FV infection, NKT cells showed direct cytotoxic activity, but no increased production of pro-inflammatory cytokines. Thus, the antiviral effect of these cells in FV-infected mice was most likely mediated by direct target cell killing and not by cytokine-induced activation of other effector cells. If the enhanced production of anti-inflammatory cytokines by NKT cells after FV infection counter-regulates immunopathology, as reported for the IAV model and SIV-infected AIDS-resistant sooty mangabeys, remains to be investigated in future studies.

Diverse immunoregulatory functions of NKT cells can be classified by phenotypic differences based on their CD4 and CD8 expression or by the absence of both molecules (DN) [1, 5]. In humans, CD4^−^ NKT cells reveal a rather cytolytic function and a Th1-biased cytokine profile while CD4^+^ NKT cells produce high levels of Th2 and also Th1-associated cytokines and exhibit immunoregulatory functions [36, 37]. During HIV and SIV infection, the CD4^+^ NKT cell subset was depleted, which was inversely correlated with viral loads [21, 22, 38] whereas others did not detect any correlations between NKT cell depletion and viral set points [30, 39]. During FV infection we did not detect a depletion of CD4^+^ NKT cells probably due to the fact that FV mainly infects erythroid precursor cells as well as granulocytes and B cells [17]. Similar to other studies we found that CD4^+^ NKT mainly produced Th2 cytokines, whereas the DN NKT cell subset expressed markers associated with immune activation and cytotoxicity. Therefore, the anti-retroviral activity of NKT cells during FV infection is most likely mediated by the DN NKT cell sub-population.

Immunotherapies targeting NKT cells as effectors aim at increasing NKT cell numbers or enhancing their effector functions. For the stimulation of NKT cells in SIV-infected macaques the exact protocol is of crucial importance for the proper initiation of NKT cell responses [40]. Treatment protocols from mouse experiments were not successful for the activation of NKT cells in humans and macaques [41]. Recently it was demonstrated that the administration of αGalCer to macaques infected with SIV resulted in an initial transient decline of NKT cell frequencies followed by an NKT cell expansion at six to nine days post αGalCer therapy [40]. Nevertheless, αGalCer was able to efficiently activate NKT cells in SIV-infected macaques [41]. In acutely FV-infected mice, the activation of NKT cells with αGalCer was associated with increased NKT cell numbers in the bone marrow and slightly in the spleen, better activation, and improved antiviral responses of NKT cells. Stimulation of FV-infected animals with αGalCer resulted in a significantly increased FasL expression on NKT cells, which was not seen in naïve mice stimulated with αGalCer. Therefore, αGalCer treatment might be an interesting new immunotherapy against retroviral infections. Interestingly, αGalCer was also tested as a mucosal adjuvant against genital herpes [42]. Here, immunization with HSV-2 glycoprotein D in combination with αGalCer improved the IgG antibody response and resulted in complete protection against vaginal HSV-2 challenge [42]. In Hepatitis B virus (HBV) infection, NKT cells were shown to be initially activated and contribute to the antiviral immune response by promoting adaptive immune responses [43]. Independently of T and B cells, stimulation of NKT cells with αGalCer abolished viral replication and increased concentrations of IFNγ and type I IFNs in HBV-transgenic mice were detected [44]. However, type I IFN responses do not play a critical role in the FV model because they are actively suppressed by the virus [45, 46]. In hepatitis virus infections NKT cells also seem to have opposing effects on pathogenesis. Beside the positive effects of activated hepatic NKT cells in preventing acute liver injury, inflammation and fibrosis, other studies demonstrated that NKT cells may also contribute to hepatic injuries in an FasL-dependent damage of hepatocytes [47, 48]. Furthermore, the excessive activation of NKT cells can result in accelerated liver damage [48, 49]. Thus, activation of hepatic NKT cells was not only associated with beneficial effects but also with impaired liver regeneration in HBV-transgenic mice [50]. In the FV model, αGalCer therapy had a beneficial effect on the course of infection, but important aspects of immunopathology have to be carefully considered for every pathogen when augmenting NKT cell responses.

In this report, we describe the impact of NKT cells on the control of an acute retroviral infection. Stimulation of NKT cells with αGalCer improved their anti-retroviral potential, which might be an interesting new approach for immunotherapy of acute virus infections.

## Methods

### Mice and FV infection

Seven to ten weeks old female inbred C57BL/6 (B6, Harlan Laboratories, Germany) were used for the experiments. All mice were treated in accordance with the regulations and guidelines of the institutional animal care and use committee of University of Duisburg-Essen. The FV stock used in these experiments was FV complex containing B-tropic Friend murine leukemia helper virus and polycythemia-inducing spleen focus-forming virus. The stock was prepared as a 15% spleen cell homogenate from BALB/c mice infected 14 days previously with 3000 spleen focus-forming units (SFFU). Mice were injected intravenously with 0.1 ml phosphate-buffered saline containing 40,000 SFFU of FV. The virus stock did not contain lactate dehydrogenase-elevating virus. Mice were sacrificed 3 dpi by cervical dislocation and spleen and bone marrow (two legs) were harvested.

### IC assay

Infectious centers (IC) were detected by tenfold dilutions of single-cell suspensions of splenocytes and bone marrow cells onto Mus dunnis cells. Co-cultures were incubated for three days, fixed with ethanol, stained with F-MuLV envelope-specific monoclonal antibody 720 and developed with peroxidase-conjugated goat anti-mouse antibody and aminoethylcarbazol for the detection of foci.

### Flow cytometry

Cell surface staining was performed for 15 min in the dark using PBS. The exclusion of dead cells was achieved using Zombie UV dye (BioLegend). Cells were stimulated with Ionomycin (500 ng/ml), PMA (25 ng/ml), Monesin (1×, BioLegend) and Brefeldin A (2 μg/ml) diluted in IMDM buffer and incubated for 3 h at 37 °C to detect cytokines and FasL expression. For intracellular stainings BD Cytofix/Cytoperm Fixation/Permeabilization kit was used. Surface and intracellular stainings were performed using following antibodies: CD3 (17A2, eBioscience), CD43 (1B11, BioLegend), CD49b (Dx5, eBioscience), CD69 (H1.2F3, eBioscience) CD86 (GL1, BioLegend), CD107a (ID4B, BioLegend), FasL (MFL3, BD Pharmingen), IFNγ (XMG1.2, eBioscience), IL-10 (JES5-16E3, eBioscience) IL-13 (eBio13A, eBioscience), NK1.1 (PK136, eBioscience), and TNFα (MP6-XT22, BioLegend).

### In vitro cytotoxicity assay

FBL-3 tumor cells were cultured in RPMI plus 1% Penicillin/Streptomycin and 10% FBS. In vitro cytotoxicity assay was performed using 1 × 10^4^ CFSE stained FBL-3 tumor cells and 25 × 10^4^ isolated NKT cells from the spleen and the bone marrow of naive or FV-infected mice. The assay was performed in 96-well U-bottom plates and co-incubation took place for 24 h in a humidified 5% CO_2_ atmosphere at 37 °C. Cells were washed once, stained for fixable viability dye (FVD, eBioscience) to exclude dead cells and analyzed by flow cytometry.

### NKT cell stimulation and isolation

At day 0 of FV infection, NKT cells were stimulated by i. p. application of 2 µg chemically synthesized αGalCer (KRN7000, Cayman Chemical Company) diluted in PBS. For isolation of NKT cells, CD3^+^ cells were isolated with MagniSort^®^ Mouse CD3 Positive Selection Kit (eBioscience) and cells were sorted for NK1.1^+^ cells. For transfer experiment, 1 × 10^5^ NKT cells per mouse were diluted in PBS and injected i.v. at the day of FV infection.

### Statistical analyses

Statistical analyses and graphical presentations were computed with Graph Pad Prism version 6. Statistical differences between two different groups were determined by the Mann–Whitney test. Differences between three groups were analyzed by Kruskal–Wallis test. Outliers were identified with the Rout method.

## Additional files

**Additional file 1: Figure S1.** Activation and effector functions of NKT cells during early FV infection. Splenocytes were isolated from FV-infected mice (3 dpi) and analyzed using flow cytometry. Representative histograms of the NKT cell activation (CD69, CD86, CD43) and effector functions (CD107a, FasL) are shown.

**Additional file 2: Figure S2.** Type I NKT cells and αGalCer therapy during the acute FV infection. Mice were infected with FV and sacrificed at 3 dpi. As control group non-infected mice were used. Single cell suspensions were prepared from the bone marrow and spleens of mice. Representative histograms for the identification of NKT cells and invariant NKT cells of a FV-infected mouse are shown in A. Activation of invariant NKT cells were analyzed in both organs by the measurement of early activation marker CD69 (B). Six animals per group out of two experiments were used for analysis. Statistically significant differences between groups were analyzed with the Mann–Whitney test and are indicated by single asterisk for *p* < 0.05. Representative histograms of NKT cells (CD3^+^NK1.1^+^) and type I NKT cells (CD3^+^ αGalCer pre-loaded CD1d tetramer^+^ NK1.1^+^) from naïve, FV-infected and FV-infected plus αGalCer-treated mice are shown in C. Activation, IFNγ and TNFα production of NK cells (CD3^–^CD49b^+^NK1.1^+^) is shown in D for groups of naïve, FV-infected and FV-infected plus αGalCer-treated mice. Data were collected from at least four independent experiments. At least seven animals per group were used for analysis. Mean (±SEM) values of percentages are indicated by bars. Statistically significant differences between groups were analyzed with the Kruskal–Wallis test and are indicated by single asterisk for *p* < 0.05 and triple asterisk for *p* < 0.001.
