# Supplementary figures and images for: Natural killer T cells contribute to the control of acute retroviral infection
Source: Retrovirology. 2017 Jan 26;14:5. doi: 10.1186/s12977-017-0327-8 (PMC5267384; doi:10.1186/s12977-017-0327-8)

## Slide 1
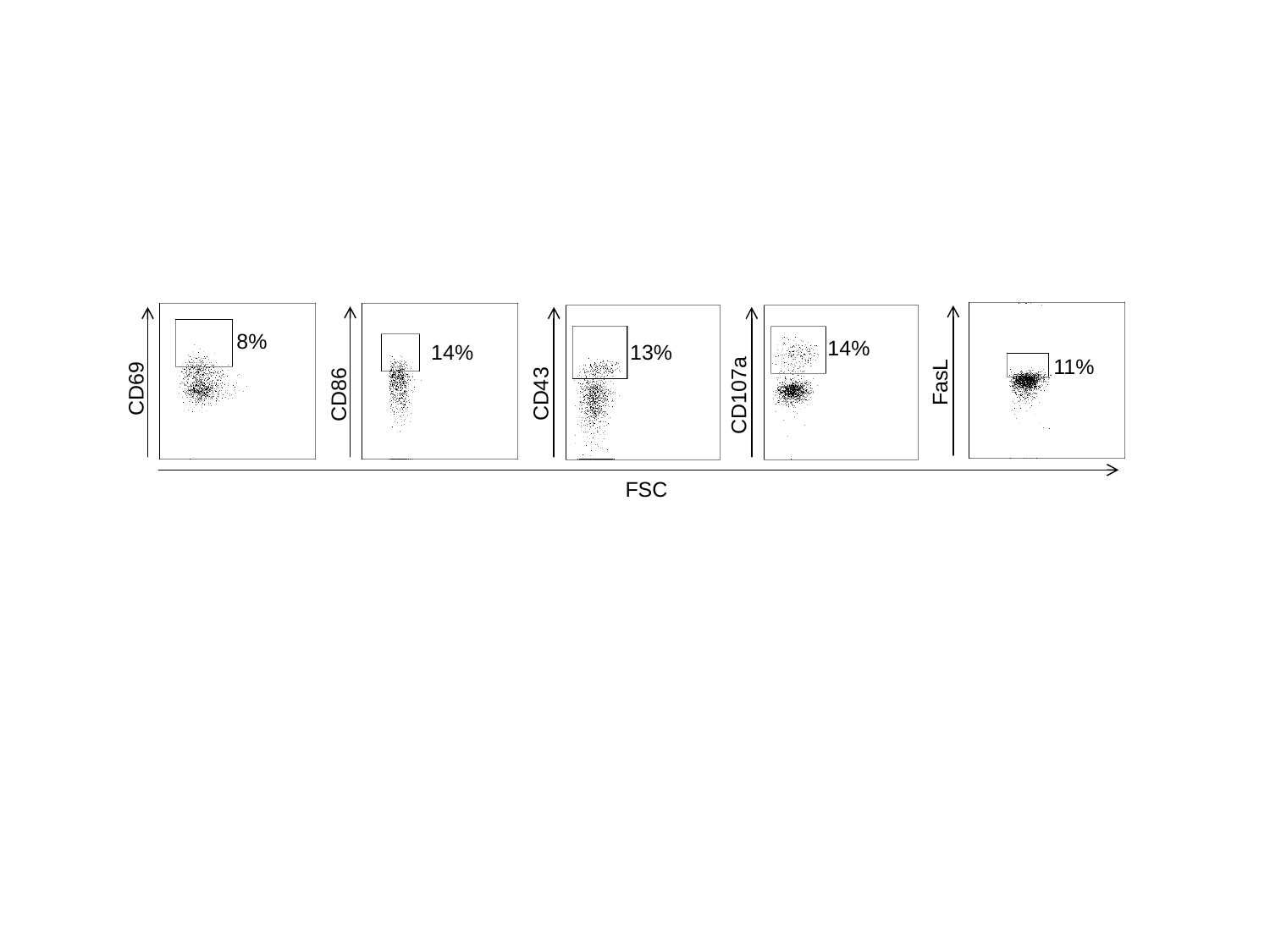

8%
14%
CD43
13%
14%
FasL
CD69
11%
CD86
CD107a
FSC

Supplement: Supplementary file 1 — Additional file 1: Figure S1. Activation and effector functions of NKT cells during early FV infection. Splenocytes were isolated from FV-infected mice (3 dpi) and analyzed using flow cytometry. Representative histograms of the NKT cell activation (CD69, CD86, CD43) and effector functions (CD107a, FasL) are shown. [file 12977_2017_327_MOESM1_ESM.pptx]
