# Supplementary material for: Natural killer T cells contribute to the control of acute retroviral infection
Source: Retrovirology. 2017 Jan 26;14:5. doi: 10.1186/s12977-017-0327-8 (PMC5267384; doi:10.1186/s12977-017-0327-8)
Supplement: Supplementary file 2 — Additional file 2: Figure S2. Type I NKT cells and αGalCer therapy during the acute FV infection. Mice were infected with FV and sacrificed at 3 dpi. As control group non-infected mice were used. Single cell suspensions were prepared from the bone marrow and spleens of mice. Representative histograms for the identification of NKT cells and invariant NKT cells of a FV-infected mouse are shown in A. Activation of invariant NKT cells were analyzed in both organs by the measurement of early activation marker CD69 (B). Six animals per group out of two experiments were used for analysis. Statistically significant differences between groups were analyzed with the Mann–Whitney test and are indicated by single asterisk for p < 0.05. Representative histograms of NKT cells (CD3+NK1.1+) and type I NKT cells (CD3+ αGalCer pre-loaded CD1d tetramer+ NK1.1+) from naïve, FV-infected and FV-infected plus αGalCer-treated mice are shown in C. Activation, IFNγ and TNFα production of NK cells (CD3–CD49b+NK1.1+) is shown in D for groups of naïve, FV-infected and FV-infected plus αGalCer-treated mice. Data were collected from at least four independent experiments. At least seven animals per group were used for analysis. Mean (±SEM) values of percentages are indicated by bars. Statistically significant differences between groups were analyzed with the Kruskal–Wallis test and are indicated by single asterisk for p < 0.05 and triple asterisk for p < 0.001. [file 12977_2017_327_MOESM2_ESM.pptx]

## Slide 1
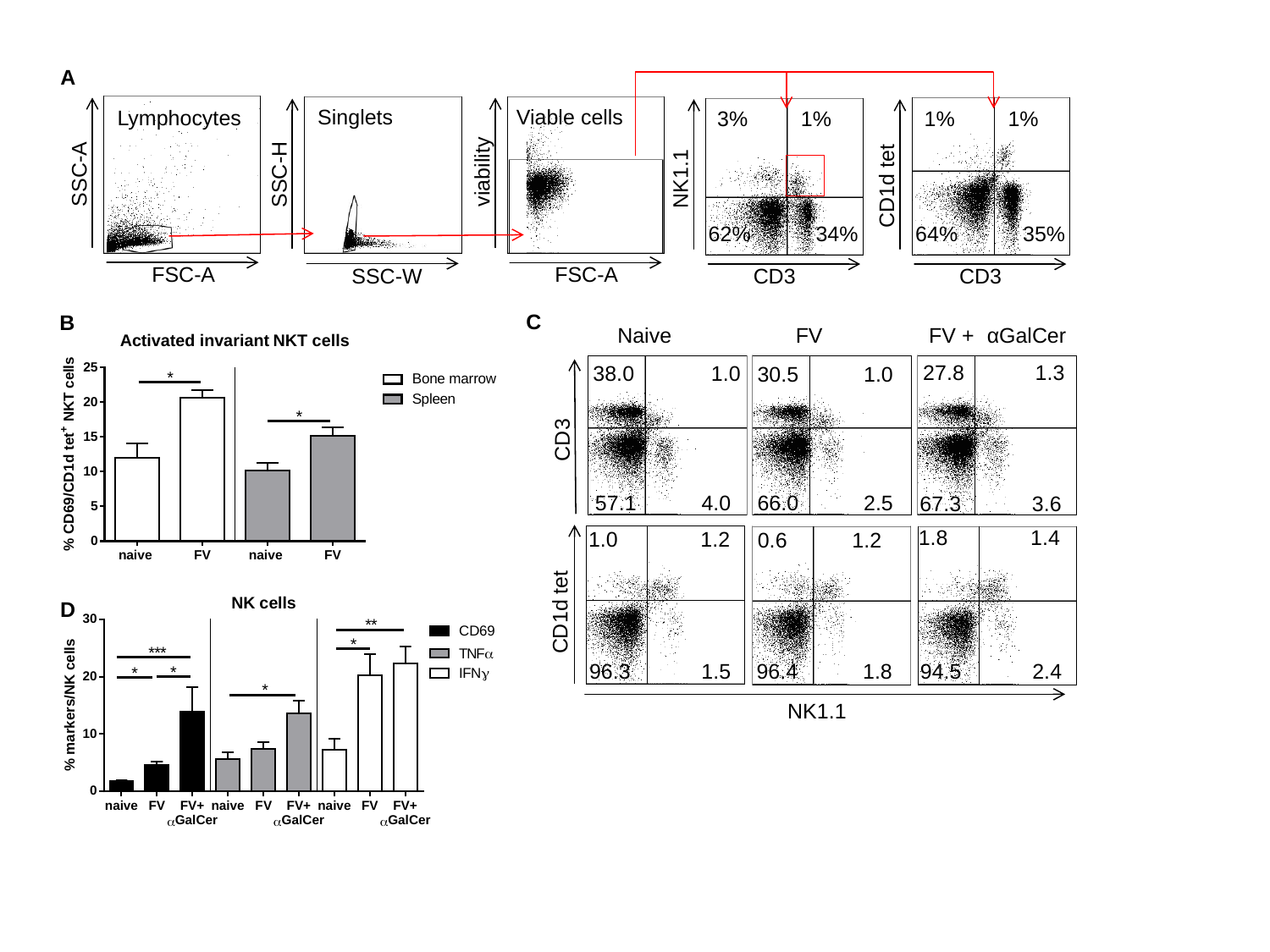

Singlets
Viable cells
Lymphocytes
1% 1%
3% 1%
CD1d tet
SSC-A
viability
SSC-H
NK1.1
64% 35%
62% 34%
FSC-A
FSC-A
SSC-W
CD3
CD3
Naive FV FV + αGalCer
27.8 1.3
38.0 1.0
30.5 1.0
CD3
57.1 4.0
66.0 2.5
67.3 3.6
1.8 1.4
1.0 1.2
0.6 1.2
CD1d tet
96.4 1.8
96.3 1.5
94.5 2.4
NK1.1
